# Draft Genome of *Frankia* sp. Strain R82, an Isolate from Root Nodules of Myrica gale

**DOI:** 10.1128/mra.00925-22

**Published:** 2022-10-26

**Authors:** Philippe Normand, Petar Pujic, Danis Abrouk, Spandana Vemulapally, Trina Guerra, Camila Carlos-Shanley, Dittmar Hahn

**Affiliations:** a Université Claude-Bernard Lyon 1, Université de Lyon, UMR 5557 CNRS Ecologie Microbienne, Villeurbanne, France; b Texas State University, Department of Biology, San Marcos, Texas, USA; Wellesley College

## Abstract

The *Frankia* sp. strain R82 genome is described as representative of a novel candidate species within *Frankia* cluster 1, as indicated by average nucleotide identity (ANI) analyses, with its closest relatives being Frankia nodulisporulans AgTrs and strains Ag45/Mut15 and AgPM24 (86% identity).

## ANNOUNCEMENT

The genus *Frankia* represents nitrogen- and non-nitrogen-fixing actinobacteria that form root nodules in symbiosis with woody plants (1, 2) or occur saprophytically in rhizospheres and soil (3). Recently, several species were described, with increasing species diversity discovered within currently delineated subunits, that is, clusters 1 to 4, with cluster 1 containing isolates infective to *Alnus*, *Myrica*, and *Casuarina* (4).

Here, we describe the draft genome of *Frankia* sp. strain R82 (LLR161101) obtained from the Agricultural Research Service Culture Collection (Peoria, IL, USA) deposited under NRRL B-16413. The strain was isolated from root nodules of Myrica gale from Black Creek National Refuge, Vermont, United States (44°50′N, 73°08′W) in 1980 (5). This strain was kept at −80°C in 20% glycerol in water (vol/vol) since 2010 and grown in defined propionate medium at 30°C for 15 days (6). Cells were centrifuged (15,000 × *g*, 5 min), sonicated (10 s at 20% output in S-450 Sonifier; Branson Ultrasonics, Danbury, CT), and DNA extracted using SurePrep soil DNA isolation kits (Fisher Scientific, Houston, TX). The DNA concentration was measured with a Qubit 2.0 fluorometer (Life Technologies, Carlsbad, USA). The DNA was moderately fragmented to 1,000 nucleotides (nt), and the libraries were prepared for sequencing using standard protocols for Illumina tagmentation and a NextSeq 2000 machine (2 × 150 bp) at the Microbial Genomics Sequencing Center (Pittsburgh, PA, USA) (6, 7). The total number of reads was 7,362,308, and the average length was 147.8 bp. Sequence reads were filtered, quality controlled, and trimmed using fastp v0.23.2 with default settings (8), and reads with average %GC of <54 were removed (6). The genome was assembled using SPAdes v3.13.0 (9) and quality checked with QUAST v5.2.0 (10). CheckM v1.0.18 with default values in the lineage workflow (lineage_set) was finally used to assess completeness and coverage (11), and PGAP v6.1 (12) was used to annotate; the *N*_50_ value was 37,329. The average nucleotide identity (ANI) (13) of the assembled genome with *Frankia* type strains of all described species and other selected genomes was determined using Pyani v0.2.11 with b (Blast) setting (14; https://pyani.readthedocs.io).

CheckM scores of 98.5% indicated that the genome of strain R82 was nearly complete, and the contamination index of 1.37 demonstrated the culture was pure. The genome size was 6.89 Mb with a GC of 70.95%, and *N*_50_ was 37,329 nt with 370 contigs, with the largest being 180,920 nt. ANI analysis suggests a new species in cluster 1, with the closest relatives being Frankia nodulisporulans AgTrs and strains Ag45/Mut15 and AgPM24, all with 86% sequence identity, followed by strain AiPa1 with 83% and other cluster 1 strains with 80% similarity, including Frankia alni, Frankia torreyi, Frankia canadensis, Frankia alpina, and Frankia casuarinae with 79%. ANI values were lower for representatives of cl2 (76 to 77%), cl3 (77%), and cl4 (76%).

### Data availability.

Raw reads were deposited to NCBI (SRA accession number SRX17215055). The genome sequence was deposited at GenBank (accession number JAMQQG000000000). Libraries and sequencing were done at the Microbial Genomics Sequencing Center.
